# One-Year Echocardiographic Follow-Up in Transthyretin Cardiac Amyloidosis: Impact of Tafamidis Treatment

**DOI:** 10.3390/jcm14051538

**Published:** 2025-02-25

**Authors:** Davide Restelli, Céline Van Wallendael, Nils De Marneffe, François Damas, Raluca Dulgheru, Patrizio Lancellotti

**Affiliations:** 1 Department of Cardiology, Valvular Diseases and Cardiomyopathy Clinic, University of Liège Hospital—CHU Sart Tilman, 4000 Liège, Belgium; celine-vw@hotmail.com (C.V.W.);; 2 GIGA Cardiovascular Sciences & Metabolism, University of Liège, 4000 Liège, Belgium

**Keywords:** cardiac amyloidosis, Tafamidis, echocardiography

## Abstract

**Background/Objectives**: Cardiac amyloidosis (CA) is a rare and severe multisystem disorder, associated with an average survival of approximately five years. Recently, Tafamidis has emerged as a promising treatment for transthyretin-related CA. This retrospective study aimed to assess disease progression through echocardiographic findings in patients with transthyretin-related CA, with a specific focus on evaluating the impact of Tafamidis in a cohort managed at our Cardiomyopathy Clinic. **Methods**: A total of 39 patients were included, of whom 28 received Tafamidis treatment, while 11 did not. Clinical, electrocardiographic, echocardiographic, biological, and other imaging data were collected at diagnosis. Comprehensive echocardiographic data were collected every six months over a two-year period (2021–2023). **Results**: At 1-year follow-up, the Tafamidis-treated cohort demonstrated stable global systolic and diastolic function. Left ventricular (LV) global longitudinal strain (GLS) and global work index (GWI) showed minimal change (GLS −12.9% (−15.6; −10.7) vs. −13.0% (−14.0; −10.7), *p* = 0.054; GWI 1113 mmHg/% (963; 1301) vs. 1208 mmHg/% (850; 1420), *p* = 0.054), and there was no significant increase in indexed LV mass (135.0 g/m^2^ (118.0; 167.0) vs. 148.0 (128.0; 173.0), *p* = 0.25). Similarly, valvular heart disease severity remained unchanged. Longitudinal analysis using generalized linear mixed models further confirmed the stability of echocardiographic parameters over the 2-year follow-up period. Systolic function metrics, including LV ejection fraction (slope: −0.0098 ± 0.011, *p* = 0.38) and GLS (slope: 0.0036 ± 0.0041, *p* = 0.39) showed no significant decline. Diastolic function assessed through E/A ratio (slope: −0.0007 ± 0.0013, *p* = 0.59) and E/e’ (slope: −0.0042 ± 0.0073, *p* = 0.57) also remained stable. Indexed LV mass exhibited no significant progression (slope: 0.050 ± 0.061, *p* = 0.41). These findings were consistent across the various subgroups. **Conclusions**: Tafamidis appears to effectively stabilize transthyretin-related CA, limiting disease progression over the follow-up period.

## 1. Introduction

In recent years, interest in cardiac amyloidosis (CA) among healthcare professionals has grown significantly. Therapeutic advances and the emergence of targeted treatments for transthyretin-related cardiac amyloidosis (ATTR-CA) have markedly improved patient outcome [1,2,3,4,5]. Since 2020, Tafamidis has been approved for the treatment of both wild-type and hereditary ATTR-CA [6]. This oral stabilizer of the transthyretin tetrameric protein prevents its dissociation into monomers or oligomers, which are key contributors to amyloid deposition. The phase III multicenter ATTR-ACT trial demonstrated the efficacy of Tafamidis in managing cardiac involvement and reported a 30% reduction in overall mortality after 30 months compared to placebo, with survival curves diverging as early as the 18th month of treatment [7]. Additionally, a significant reduction in the risk of cardiovascular hospitalization was observed, along with improvements in quality of life and functional decline as early as six months. The efficacy of Tafamidis appears to remain consistent even over an extended follow-up period [8]. However, Tafamidis appears slightly less effective in patients with advanced heart failure and dyspnea on minimal exertion (New York Heart Association—NYHA class III). Managing these patients remains challenging, particularly in determining reliable parameters to evaluate treatment response and monitor disease progression. Echocardiography continues to be the cornerstone of monitoring, with several markers proposed for detecting adverse progression function [9]. This study aimed to evaluate the impact of Tafamidis on echocardiographic parameters in transthyretin-related CA, focusing on its role in stabilizing cardiac structure and function and identifying reliable markers for treatment monitoring.

## 2. Research Protocol and Methods

This was a retrospective, single-center study conducted over two years, from January 2021 to December 2023 at the Centre Hospitalier Universitaire (CHU) of Liège, Belgium. We analyzed baseline demographic, clinical, biological, and instrumental data from patients diagnosed with ATTR-CA. The progression of echocardiographic and biological parameters was then evaluated, comparing patients treated with Tafamidis to those untreated, to assess its effectiveness. Subgroup analyses were conducted based on indexed left ventricular (LV) mass, age, LV ejection fraction (EF), and NYHA functional class. Our data were compared with published studies, including the ATTR-ACT trial and research on Tafamidis’ impact on echocardiographic parameters such as global longitudinal strain (GLS) and myocardial work [7,10,11]. For data collection, we used the OmniPro medical software version 2.26.6 (Zorgi SA, Bruxelles, Belgium) and the Reseau de Santé Wallon digital medical record (Fédération Régionale des Associations de Télématique Médicale de Wallonie, Liège, Belgium). The study adhered to the principles outlined in the Declaration of Helsinki and received approval from the Ethics Committee of Liege University Hospital (protocol code: 2023/3039).

### 2.1. Study Population

We studied patients with confirmed ATTR-CA by scintigraphy or biopsy and who have been followed at the Cardiomyopathy Clinic at CHU of Liège, Belgium. Exclusion criteria were the absence of informed consent, mixed ATTR and AL amyloidosis or undefined cardiac amyloidosis, inadequate echocardiographic image quality for reliable measurements, and clinical conditions or comorbidities limiting life expectancy to less than one year.

### 2.2. Data Collected at Diagnosis

#### 2.2.1. Clinical and Instrumental Data

Demographic data, medical history (including carpal tunnel syndrome, lumbar spinal stenosis, biceps tendon rupture, orthostatic hypotension, and peripheral neuropathy), clinical data, and blood test analyses (including N-terminal pro b-type natriuretic peptide (NT-proBNP), and urinary or blood light chains) were recorded. Ongoing medical therapy was documented. Electrocardiograms (ECGs) were evaluated for conduction disturbances, pseudo-infarct patterns, microvoltage, or atrial fibrillation. Bone scintigraphy was performed using Technetium-99m hydroxymethylene diphosphonate and only Perugini grade results 2 and 3 were considered positive for ATTR-CA, together with negative hematologic tests.

#### 2.2.2. Echocardiographic Data

We analyzed LV dimensions and mass, cardiac output, LV function (measured by LVEF, LV strain, myocardial work parameters as global work index (GWI) and global work efficiency (GWE)), left atrial (LA) dimension and function (including LA strain), diastolic function, right ventricular (RV) function (including RV strain), presence of pulmonary hypertension, and presence and grading of valvular heart disease. Images were acquired using GE Vivid E95 echographic machine (General Electric HealthCare, Chicago, IL, USA).

#### 2.2.3. Tafamidis Eligibility

The eligibility criteria for Tafamidis were consistent with Belgium’s reimbursement criteria, which are determined by the severity of symptoms. Reimbursement is limited to patients in NYHA class I and II, excluding those in NYHA class III and IV.

#### 2.2.4. Patient Follow-Up

Six-month follow-up data were collected retrospectively for a total time range of two years. The patients were followed for a median (Q1; Q3) of 67 weeks (40; 101): duration (Q1; Q3) was 52 weeks (30; 134) in the group without Tafamidis and 68 weeks (44; 90) in the group with Tafamidis, without significant difference between the two groups (*p* = 0.71). A total of 5 patients (4 without Tafamidis and 1 with Tafamidis) died during the follow-up period because of cardiovascular causes. Considering the availability of echocardiographic data, we conducted a comprehensive comparison at one year, and then used generalized linear mixed models (GLMM) to assess broader trends in selected parameters over the entire follow-up period.

### 2.3. Statistical Analyses

Quantitative variables are described using means and standard deviations (mean ± SD) or medians and interquartile ranges (Q1; Q3). Qualitative variables are described using frequency tables (counts and percentages). Quantitative variables are compared using analysis of variance (ANOVA) or Kruskal–Wallis non-parametric tests, depending on whether their distribution is Gaussian or not. Qualitative variables are compared using Fisher’s exact test. The change between the initial value (Day 0) and the one-year value (Day 360) for each quantitative variable in each group is analyzed using the non-parametric signed-rank test for paired observations. Comparisons of changes between groups were made using the Kruskal–Wallis test. Comparisons were not calculated when the number of observations was insufficient (arbitrarily set at <4 patients). For each parameter, overall progression was also analyzed using generalized linear mixed models (GLMM), which allow for time-based analysis using all available observations (from Day 0 to Day 720). In these models, a ‘Group = Tafamidis’ parameter was added to compare the two groups overall, and the ‘Group × Time’ parameter allows analysis of whether changes over time differ between the two groups. For qualitative variables, changes over time (Day 0 to Day 360) were compared using the McNemar test. Analyses were conducted on the maximum available data, and missing values were not imputed. Results were considered statistically significant at a 5% threshold (*p* < 0.05). The analyses were performed using SAS software version 9.4 (SAS Institute, Cary, NC, USA). To compare our results with reference studies, a Wilcoxon signed-rank test using PASS software version 11 (NCSS LCC, East Kaysville, UT, USA) was performed.

## 3. Results

### 3.1. Description of the Population at Baseline

A total of 39 patients were included, all newly diagnosed with ATTR-CA and not receiving any disease-specific therapy. Of these, 28 were deemed eligible for Tafamidis treatment and initiated therapy with Tafamidis 61 mg once daily, while the remaining 11 did not receive any disease-specific treatment.

### 3.2. Clinical Data

Table 1 summarizes the principal clinical characteristics of patients at baseline, comparing those treated with Tafamidis to those who were not. Patients in the non-Tafamidis group were, on average, older than those in the Tafamidis group (*p* = 0.0002), and had higher NYHA functional class score (*p* = 0.016). The full Table S1 is available in the Supplementary Material.

### 3.3. Echocardiographic Data

Table 2 presents baseline transthoracic echocardiographic data. Patients eligible for Tafamidis exhibited a more negative GLS compared to the non-treated group, while LVEF was similar between groups. Tricuspid Annular Plane Systolic Excursion (TAPSE) and tissue Doppler e’ were higher in the Tafamidis group. The Tafamidis treated group seemed to have more aortic regurgitation (AR) than the non-treated group, but AR severity was negligible in both groups. Conversely, the non-treated group demonstrated higher transaortic velocity and gradient, though these values did not meet criteria for aortic stenosis. The full Table S2 is available in the Supplementary Material.

Follow-Up Echocardiography

### 3.4. Evolution of LV Systolic Function Parameters

In the group without Tafamidis, no significant changes in LV systolic function parameters are observed at one year (Table 3). Unfortunately, the number of patients is too small to draw conclusions with sufficient statistical power. While the medians suggest a trend toward deterioration, this cannot be definitively asserted. In the group with Tafamidis, LVEF significantly decreases between baseline and Day 360 (*p* = 0.0084) (Table 3), but does not show a significant decline when considering data over the entire follow-up period (Table 4). GLS average is lower in Tafamidis group (*p* = 0.012) but without significant evolution at one year and over the entire follow-up (Table 3 and Table 4). There were no significant changes at one year and over the entire follow-up for GWI and GWE, even if a better tendency in the treated group was observed (Table 3 and Table 4). We do not identify any significant differences between the groups regarding changes in global LV systolic function parameters at one year and in the entire follow-up period, even considering that the mean GLS is overall more negative in the group with Tafamidis. This finding should also be tempered by the small number of patients in the group without Tafamidis and the duration of follow up.

### 3.5. Comparison of Our Results with Reference Studies

**Figure 1:**
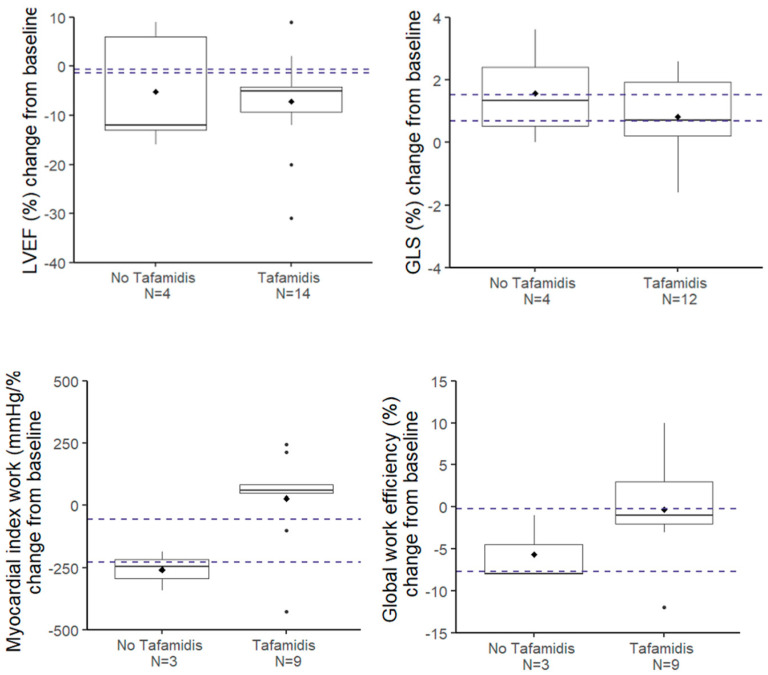
Evolution at 1 year of the parameters of the left ventricular systolic function. The dotted horizontal lines represent the 95% confidence interval of the evolution at 1 year in the group without Tafamidis according to the article by Giblin et al. [11].

A lack of statistically significant progression may be due to an insufficient number of observations and so a lack of statistical power, especially in the untreated group. With regard to GLS, reference articles [10,11] will help us establish the hypotheses needed to calculate the number of patients required to achieve sufficient power to detect a clinically significant change in GLS after one year. With a sample size of N = 12 patients, we can detect a difference of 1% (with a standard deviation of 1) at an alpha level of 0.05 with 85% power. This power calculation indicates that the sample size of our Tafamidis-treated group is sufficient to detect a 1% change in GLS after one year. Thus, we can conclude that there is no significant progression in GLS in this subgroup after one year (Figure 1). A similar reasoning can be made for LVEF (slightly worsened), GWI (slightly improved), and GWE (unchanged), as shown in Figure 1. Globally, we can consider that the LV function does not change after one year. However, in the subgroup without Tafamidis, the sample size is too small to draw reliable conclusions. Therefore, the results for this subgroup, as well as comparisons between the Tafamidis-treated and untreated groups, should be interpreted carefully.

### 3.6. Evolution of Other Echocardiographic Parameters

The analysis of cardiac function parameters over one year revealed no statistically significant changes in LV mass in either the Tafamidis or non-Tafamidis groups, as shown in Table 5. This result should be interpreted with caution due to the small sample size of the non-treated group, which limits the statistical power of comparisons. In any case, no significant differences were found between the groups and this trend was confirmed in the overall evolution (Table 5 and Table 6). RV function parameters (dimension, TAPSE, S’) also showed no significant changes over one year in either group, without statistically significant difference between the groups regarding changes after one year, though TAPSE tended to be higher in the Tafamidis group without significant progression over time (Table 5). This tendency was confirmed in the overall observed period analysis (Table 6). More, pulmonary acceleration time is generally higher in the Tafamidis group, though there is no difference in its evolution over time. Again, these results should be interpreted with caution due to the small sample size of the non-treated group. Left atrial function parameters (surface, volume, and strain) exhibited no statistically significant changes at one year in the Tafamidis group, and insufficient data in the non-Tafamidis group precluded meaningful comparisons. Diastolic function parameters (E, A, E/A, e’, E/e’, Tissue Doppler parameters, left atrial volume, Tricuspid Regurgitation Pressure Gradient (TRPG)) demonstrated a non-significant evolution at one year in either group. Regarding the overall trends throughout the study period (Table S10), the Tafamidis group generally displayed higher septal and lateral e’ values without significant changes in E/e’ ratio compared to the non-Tafamidis group. A similar reasoning could be made for TRPG, lower in the Tafamidis group but without significant changes compared to the non-treated group. A non-significant trend toward a decrease in A wave velocity was observed, with a more pronounced decline in the Tafamidis group, without significant changes in E/A ratio, warranting further investigation in larger patient samples. Importantly, no deterioration in valvulopathies was observed at one year in the Tafamidis group. These findings, limited by small sample sizes, emphasize the need for larger, more comprehensive studies to better assess the potential impacts of Tafamidis on cardiac function parameters and disease progression. Full Tables S3–S10 are available in the Supplementary Material.

## 4. Discussion

### 4.1. Baseline Characteristics of Untreated and Treated Groups

In comparing the baseline populations, the untreated group appears more severely affected by amyloidosis. They were older and had a higher NYHA class compared to those receiving Tafamidis. Additionally, they exhibited lower albumin levels and elevated troponin and sodium levels. The untreated group also had a higher incidence of pathological Q-waves and microvoltages on ECG. Echocardiographic data revealed more pronounced strain alterations and lower TAPSE, indicating both left and right ventricular systolic dysfunction. Notably, although LVEF remained normal, the GLS was significantly impaired, underscoring GLS as a more sensitive metric for diagnosing and monitoring cardiac amyloidosis. Moreover, non-significant higher mean E/e’ ratios and systolic pulmonary artery pressure suggested elevated filling pressures and a higher likelihood of pulmonary hypertension. These results may not fully align with the literature [7,11], likely because our untreated patients, owing to the reimbursement criteria for Tafamidis, tend to be at a more advanced stage of the disease, with more pronounced cardiovascular compromise. In fact, in Belgium, Tafamidis is not reimbursed in patients with NYHA class ≥ III.

### 4.2. Stability of Echocardiographic Parameters in Treated Patients

Over 1-year follow-up, the Tafamidis-treated group demonstrated overall stability across key echocardiographic parameters. There were no statistically significant changes in global ventricular systolic (LVEF, GLS, GWI, and GWE) and diastolic function (e’, E/e’), LV mass, LA size, or valvular disease severity, suggesting that Tafamidis effectively halts or slows disease progression and prevents further myocardial damage in these patients. Similarly, the lack of significant changes in LV mass and LA size supports the hypothesis that Tafamidis not only stabilizes myocardial function but also mitigates structural remodeling of the heart. Overall, this stabilization is particularly significant given the progressive nature of cardiac amyloidosis, which is typically marked by worsening systolic strain, increasing myocardial thickness, and rising filling pressures over time [1,3]. Although LA strain values remained unchanged in our treated patients, prior studies have reported improvements in wild-type ATTR-CA patients in sinus rhythm [12]. The absence of similar findings in our study could be attributed to several factors, including the relatively small size of our cohort and the presence of atrial fibrillation or other rhythm abnormalities in some patients, which are known to impact LA strain values [12]. Furthermore, the duration of follow-up and baseline characteristics of the cohort, such as more advanced disease stages in some patients, may also have contributed to this difference. Focusing on LV systolic function, in both groups at baseline, the nature of ATTR-CA was reflected in lower GLS values (−12% [Q1; Q3] = −12.3; −9) compared to standard reference values (>−16%), lower GWI (1100 mmHg/% [963; 1301], reference 1896 mmHg/% ± 308), and reduced GWE (94% [88; 95], reference range 94–97%). These results are in line with data from the literature [4,7]. In patients treated with Tafamidis, LV systolic function parameters stabilized over the year. The primary aim of Tafamidis therapy is indeed to slow or stabilize disease progression [7,8,10,11,13].

Due to the small sample size regarding the untreated cohort, no conclusive statistical power could be achieved in this group. However, we referenced existing studies to approximate the natural course of untreated amyloidosis. In the ATTR-ACT study [7], GLS declined more rapidly in untreated patients over 30 months, with a decrease of 2.16% (Standard Error—SE 0.33) in the placebo arm compared to 1.46% (SE 0.28) in the Tafamidis-treated cohort. However, it is not possible to extrapolate these results due to the pooled nature of the data. The Apollo study [14] found a GLS deterioration of 1.46% in the placebo group during a follow up of 18 months. In a separate 12-month study [15], GLS declined by 1.2% in the untreated group. A 2022 study by Giblin et al. [11] reported greater deterioration in untreated patients’ GLS, GWI, and GWE compared to treated patients over a 12-month period. In light of the natural progression of cardiac amyloidosis, our findings support previous studies showing a stabilization of GLS, GWI, and GWE in Tafamidis-treated patients [2,7,8,10,11]. In our study, Tafamidis demonstrated efficacy regardless of age and indexed left ventricular mass. Although not statistically conclusive, median values suggest less pronounced decline in the treated group versus untreated on parameters like GLS (0.7% vs. 1.3%), GWI (+60 mmHg% vs. −247 mmHg%), and GWE (−1% vs. −8%). This is in line with data presented in the literature [16].

### 4.3. Safety Profile of Tafamidis Treatment

Among treated patients, we recorded two cases of mild dyspepsia and diarrhea and one case of transient itching, all of which resolved spontaneously without intervention, and not clearly caused by Tafamidis itself. These findings confirm the good clinical safety profile of the drug. [17]

## 5. Limitations

This study has several limitations that should be considered. The small cohort size, particularly among untreated patients, reflects the rarity of the disease and restricts the study’s statistical power and reproducibility of conclusions. Not all the echocardiographic data were available during the follow up due to sub-optimal patient compliance: this could have limited and potentially biased short-term and long-term analyses. The single-center design, though enhancing measurement reproducibility, limits the generalizability of findings to other centers. Baseline differences between the treated and untreated groups, including advanced disease in many untreated patients due to eligibility criteria to Tafamidis and unequal cohort sizes, complicate direct comparisons. These limitations underscore the need for larger, multicenter studies to validate and extend these findings.

## 6. Conclusions

This study suggests that Tafamidis may contribute to stabilizing cardiac function and structure in ATTR-CA, as reflected by the absence of significant adverse progression in key echocardiographic parameters. A strength of this study is its comprehensive echocardiographic assessment, which demonstrated stability not only in LV systolic function but also in LV mass, diastolic function, right ventricular performance, and valvular diseases during treatment. In monitoring LV function, a multiparametric echocardiographic assessment appears more reliable, enabling a comprehensive evaluation. However, considering the limited sample size of our study, larger studies are needed to determine which parameters could be most effective for patient follow-up. These results emphasize the value of advanced echocardiography in the baseline assessment and monitoring of ATTR-CA while reinforcing the importance of patient compliance and further research to better understand disease progression and treatment effects.

## Figures and Tables

**Table 1:** Clinical characteristics of patients at baseline.

	No Tafamidis (N = 11)	Tafamidis (N = 28)	Comparison *p*-Value
Gender, Male	8	22	0.69
Age (Years)	87.4 ± 3.6	79.5 ± 5.9	0.0002
≥80 years	11	15	
BMI (kg/m^2^)	25.7 ± 4.2	27.0 ± 3.5	0.35
SBP (mmHg)	123.6 ± 15.0	123.1 ± 13.3	0.92
HR (bpm)	72.2 ± 19.4	75.0 ± 15.1	0.63
Atrial fibrillation	7	17	1.0
Smoker	3	8, N = 27	1.0
Dyslipidemia	9	22	1.0
Diabetes (Type II)	2	8	0.69
Hypertension	6	16	1.0
NYHA			0.0016
I	0	2	
II	0	22	
III	7	4	
IV	4	0	
Coronaropathy > 50%	4	10	1.0
Pulmonary hypertension	6	13	0.73
Hospitalization for acute heart failure	4	8	0.71
Length of stay (days)	6.5 (6; 9)	5.5 (4; 7)	0.26
Carpal tunnel	2	8	0.69
Lower limb oedema	1	3	1.0
Orthostatic hypotension	1	6	0.65

BMI: Body Mass Index; SBP: Systolic Blood Pressure; HR: Heart Rate; NYHA: New York Heart Association. Results are expressed as number (percentage), mean ± standard deviation or median (Q1; Q3); and, respectively, compared using Fisher’s exact test, ANOVA or the Kruskal–Wallis non-parametric test.

**Table 2:** Data from transthoracic echocardiography at baseline.

	No Tafamidis (N = 11)		Tafamidis (N = 28)		Comparison
	N Non Missing	Results	N Non Missing	Results	*p*-Value
Telediastolic volume (mL)	11	73 (60; 100)	28	92 (68; 107)	0.26
SVi (mL/m^2^)	9	34 (28; 43)	26	35 (30; 41)	0.79
LVEF (%)	11	54 (48; 60)	28	56 (51;62)	0.16
GLS average (%)	9	−10.2 (−12.3; −10.1)	27	−13.9 (−15.9; −11.2)	0.019
Global work index (mmHg/%)	7	1037 (780; 1160)	25	1219 (963; 1347)	0.16
Global work efficiency (%)	7	90 (85; 92)	25	92 (87; 95)	0.14
LA Volume (mL/m^2^)	8	38 (32; 53)	22	48 (41;61)	0.15
LA GLS S-CT (%)	6	−2.5 (−7; −2)	14	−4 (−5; −2)	0.83
LA GLS S-R (%)	6	13 (10; 21)	14	10 (8; 13)	0.51
IVSd (mm)	11	16.0 (15.7; 18.0)	28	15.5 (13.5; 17.3)	0.20
LVEDD (mm)	11	45 (33; 50)	28	45 (39; 49)	0.79
LV mass index (g/m^2^)	11	166 (137; 183)	28	138 (120; 169)	0.055
E Velocity (cm/s)	10	84 (72; 94)	28	89 (71; 120)	0.26
E/A	6	1.2 (0.74; 1.9)	18	1.5 (0.80; 2.2)	0.35
e’ septal (cm/s)	9	4 (4; 5)	27	6 (5; 6)	0.041
e’ lateral (cm/s)	9	5 (4; 7)	27	7 (6; 9)	0.012
E/e’ mean	9	18.8 (11.0; 22.0)	27	14.0 (11.5; 16.5)	0.19
Vmax TR (m/s)	10	3.2 (2.9; 3.5)	21	2.9 (2.8; 3.0)	0.12
sPAP (mmHg)	10	53 (37; 60)	21	42 (36; 50)	0.19
RVEDD (mm)	11	38 (32; 46)	26	39 (36; 43)	0.91
TAPSE (mm)	10	15 (15; 18)	28	20 (16; 24)	0.024
S’ tricuspid (cm/s)	7	8 (7; 14)	24	11 (9; 13)	0.19
RA surface (cm^2^)	10	18.2 (15.8; 21.8)	28	20.5 (16.2; 24.0)	0.47
GLS RV G (%)	8	−9.7 (−15.6; −8.7)	17	−13.8 (−15.5; −11.1)	0.27
GLS RV FW (%)	8	−12.9 (−19.6; −11.9)	17	−16.4 (−19.9; −12.8)	0.56
AR—Grade	11		27		0.016
Trace		3		20	
Mild		7		5	
Moderate		1		2	
Severe		0		0	
AS Grade	11		27		0.070
Trace		7		23	
Mild		0		4	
Moderate		3		0	
Severe		1		0	
Peak velocity (m/s)	10	2.0 (1.6; 3.1)	27	1.4 (1.2; 1.6)	0.021
Aortic Max gradient (mmHg)	10	18.5 (10.0; 39.0)	27	7.0 (5.0; 9.6)	0.013
Aortic Mean gradient (mmHg)	10	9 (6; 20)	27	4 (3; 5)	0.0076
TVI aortic valve (cm)	10	41.0 (31.9; 57.5)	26	28.0 (23.0; 32.7)	0.0094
Velocity ratio	9	0.40 (0.29; 0.57)	26	0.70 (0.55; 0.76)	0.030
Aortic valve area (cm^2^)	9	1.8 (1.1; 2.6)	26	2.4 (2.1; 2.7)	0.15

SVi: Stroke Volume index; LVEF: Left Ventricular Ejection Fraction; GLS: Global Longitudinal Strain; LA: Left Atrium; S-CT: contractile strain; S-R: reservoir strain; IVSd: Interventricular Septum diastole; LVEDD: Left Ventricular End Dyastolic Diameter; LV: Left Ventricle; TR: Tricuspid Regurgitation; sPAP: systolic pulmonary artery pressure; RVEDD: Right Ventricular End Dyastolic Diameter; TAPSE: Tricuspid Annular Plane Systolic Excursion; RA: Right Atrium; RV: Right Ventricle; AR: Aortic Regurgitation; AS: Aortic Stenosis; TVI: Time Volume Integral; G: Global; FW: Free Wall. Results are expressed as number (percentage), mean ± standard deviation or median (Q1; Q3); and, respectively, compared using Fisher’s exact test, ANOVA or the Kruskal–Wallis non-parametric test.

**Table 3:** 1-year evolution of left ventricular systolic function parameters.

	No Tafamidis		Tafamidis		Comparison
	N	Median (Q1; Q3)	N	Median (Q1; Q3)	*p*-Value ^b^
LVEF (%)					
D0	5	55.0 (54.0; 61.0)	14	60.5 (55.0; 64.0)	
D360	5	48.0 (39.0; 60.0)	14	52.5 (45.0; 58.0)	
Change from baseline	5	−12.0 (−13.0; 6.0)	14	−5.0 (−10.0; −4.0)	0.85
*p*-value evolution ^a^		0.31		0.0084	
GLS average (%)					
D0	4	−11.2 (−12.3; −9.0)	12	−12.9 (−15.6; −10.7)	
D360	4	−9.1 (−11.0; −7.2)	12	−13.0 (−14.0; −10.7)	
Change from baseline	4	1.3 (0.35; 2.8)	12	0.7 (0.00; 2.0)	0.36
*p*-value evolution ^a^		0.25		0.054	
Global work index (mmHg/%)					
D0	3	1078 (1023; 1174)	9	1113 (963; 1301)	
D360	3	831 (680; 988)	9	1208 (850; 1420)	
Change from baseline	3	−247 (−343; −186)	9	60 (48; 84)	-
*p*-value evolution ^a^		-		0.054	
Global work efficiency (%)					
D0	3	90 (78; 92)	9	94 (88; 95)	
D360	3	82 (77; 84)	9	94 (89; 95)	
Change from baseline	3	−8 (−8; −1)	9	−1 (−2; 3)	-
*p*-value evolution ^a^		-		0.91	

LVEF: Left Ventricular Ejection Fraction; GLS: Global Longitudinal Strain. ^a^ signed rank test at the value for paired observations. ^b^ signed Kruskal–Wallis test at the *p* value.

**Table 4:** Global evolution of systolic function parameters over the entire period, evaluated using GLMM.

	Effect	Coeff. ± SE	*p*-Value
LVEF (%)	Intercept	50.6 ± 2.5	-
	Time	−0.0020 ± 0.0094	0.83
	Tafamidis	5.6 ± 3.0	0.064
	Time × Tafamidis	−0.0098 ± 0.011	0.38
GLS average (%)	Intercept	−10.7 ± 0.94	-
	Time	0.0014 ± 0.0036	0.70
	Tafamidis	−2.7 ± 1.1	0.012
	Time × Tafamidis	0.0036 ± 0.0041	0.39
Global work index (mmHg/%)	Intercept	952 ± 108	-
	Time	−0.37 ± 0.49	0.46
	Tafamidis	183 ± 121	0.14
	Time × Tafamidis	0.52 ± 0.56	0.35
Global work efficiency (%)	Intercept	88.1 ± 1.9	-
	Time	−0.0082 ± 0.0087	0.35
	Tafamidis	2.9 ± 2.1	0.19
	Time × Tafamidis	0.010 ± 0.0098	0.30

GLMM: Generalized Linear Mixed Models; LVEF: Left Ventricular Ejection Fraction; GLS: Global Longitudinal Strain.

**Table 5:** 1-year evolution of echocardiographic parameters.

	No Tafamidis		Tafamidis		Comparison
	N	Median (Q1; Q3)	N	Median (Q1; Q3)	*p*-Value ^b^
LV mass index (g/m^2^)					
D0	6	157.0 (137.0; 176.0)	15	135.0 (118.0; 167.0)	
D360	6	160.0 (148.0; 170.0)	15	148.0 (128.0; 173.0)	
Change from baseline	6	−5.5 (−25.0; 10.0)	15	4.0 (−4.0; 14.0)	0.24
*p*-value evolution ^a^		0.62		0.25	
TAPSE (mm)					
D0	4	15.0 (12.5; 16.5)	13	19.0 (17.0; 24.0)	
D360	4	17.5 (12.0; 23.0)	13	20.0 (14.0; 21.0)	
Change from baseline	4	4.5 (−0.50; 6.5)	13	−2.0 (−3.0; 0.0)	0.099
*p*-value evolution ^a^		0.38		0.077	
A Velocity (cm/s)					
D0	3	68 (38; 99)	6	73 (69; 87)	
D360	3	40 (32; 99)	6	74 (64; 87)	
Change from baseline	3	−6 (−28; 0)	6	3 (−19; 17)	-
*p*-value evolution ^a^		-		1.0	
e’ septal (cm/s)					
D0	3	4.0 (3.0; 6.0)	12	6.0 (5.5; 7.5)	
D360	3	3.0 (3.0; 4.0)	12	5.0 (5.0; 6.5)	
Change from baseline	3	−1.0 (−3.0; 1.0)	12	−1.0 (−1.0; 0.0)	-
*p*-value evolution ^a^		-		0.055	
e’ lateral (cm/s)					
D0	4	4.5 (3.5; 6.5)	13	7.0 (7.0; 9.0)	
D360	4	3.5 (3.0; 5.0)	13	7.0 (6.5; 9.0)	
Change from baseline	4	0.0 (−1.0; 1.0)	13	0.0 (−1.0; 1.0)	0.23
*p*-value evolution ^a^		0.25		0.73	
TRPG (mmHg)					
D0	5	45 (32; 50)	11	37 (31; 44)	
D360	5	41 (36; 41)	11	33 (23; 43)	
Change from baseline	5	−1 (−11; 9)	11	−3 (−20; 1)	0.50
*p*-value evolution ^a^		0.63		0.11	

LV: Left Ventricle; TAPSE: Tricuspid Annular Plane Systolic Excursion; TRPG: Tricuspid Regurgitation Pressure Gradient. ^a^ signed rank test at the value for paired observations. ^b^ signed Kruskal–Wallis test at the *p* value.

**Table 6:** Global evolution of echocardiographic parameters over the entire period, evaluated using GLMM.

	Effect	Coeff. ± SE	*p*-Value
	Intercept	172 ± 13.5	-
LV mass index (g/m^2^)	Time	−0.040 ± 0.050	0.84
	Tafamidis	−26.0 ± 15.8	0.10
	Time × Tafamidis	0.050 ± 0.061	0.41
TAPSE (mm)	Intercept	16.2 ± 1.5	-
	Time	0.0030 ± 0.0055	0.58
	Tafamidis	3.5 ± 1.8	0.050
	Time x Tafamidis	−0.0082 ± 0.0064	0.20
A Velocity (cm/s)	Intercept	72.0 ± 9.0	-
	Time	−0.066 ± 0.036	0.073
	Tafamidis	−3.3 ± 10.4	0.75
	Time × Tafamidis	0.088 ± 0.043	0.045
e’ septal (cm/s)	Intercept	4.5 ± 0.55	-
	Time	−0.0029 ± 0.0021	0.18
	Tafamidis	1.5 ± 0.62	0.015
	Time × Tafamidis	0.0011 ± 0.0024	0.65
e’ lateral (cm/s)	Intercept	5.7 ± 0.74	-
	Time	−0.0002 ± 0.0026	0.93
	Tafamidis	2.6 ± 0.85	0.0034
	Time × Tafamidis	−0.0014 ± 0.0030	0.64
TRPG (mmHg)	Intercept	40.8 ± 3.0	-
	Time	−0.0087 ± 0.010	0.40
	Tafamidis	−7.7 ± 3.7	0.037
	Time × Tafamidis	0.00052 ± 0.013	0.97

LV: Left Ventricle; TAPSE: Tricuspid Annular Plane Systolic Excursion; TRPG: Tricuspid Regurgitation Pressure Gradient; GLMM: generalized linear mixed models.

## Data Availability

The original contributions presented in this study are included in the article and supplementary materials. Further inquiries can be directed to the corresponding authors.

## Supplementary Materials

The following supporting information can be downloaded at: https://www.mdpi.com/article/10.3390/jcm14051538/s1, Table S1. Clinical characteristics of patients at baseline; Table S2. Echocardiographic data at baseline; Table S3. Evolution of left ventricular mass at 1 year; Table S4. Evolution of left ventricular mass: global evolution (GLMM); Table S5. Evolution at 1 year of right ventricular function parameters; Table S6. Evolution of right ventricular function: global evolution (GLMM); Table S7. Evolution of left atrium function at 1 year; Table S8. Evolution of diastolic function at 1 year; Table S9. Evolution of diastolic function: global evolution (GLMM); Table S10. Search for deterioration at 1 year of valvulopathies in the group with Tafamidis (N = 20 patients with values at days 0 and 360).
