# Supplementary material for: One-Year Echocardiographic Follow-Up in Transthyretin Cardiac Amyloidosis: Impact of Tafamidis Treatment
Source: J Clin Med. 2025 Feb 25;14(5):1538. doi: 10.3390/jcm14051538 (PMC11899885; doi:10.3390/jcm14051538)
Supplement: Supplementary file 1 [file jcm-14-01538-s001.zip › jcm-3431393-supplementary.pdf]

Table S1. Clinical characteristics of patients at baseline.

	No Tafamidis (N=11)	Tafamidis (N=28)	Comparison p-value
Gender, Male	8 (72.7)	22 (78.6)	0.69
Age (Years)	87.4 ± 3.6	79.5 ± 5.9	0.0002
≥ 80 years	11 (100.0)	15 (53.6)	
Height (cm)	167 ± 4.6	169 ± 7.1	0.34
Weight (kg)	72.2 ± 12.8	77.1 ± 8.7	0.17
BMI (kg/m²)	25.7 ± 4.2	27.0 ± 3.5	0.35
BSA (m²)	1.8 ± 0.15	1.9 ± 0.12	0.11
SBP (mmHg)	123.6 ± 15.0	123.1 ± 13.3	0.92
DBP (mmHg)	68.2 ± 10.8	71.6 ± 6.4	0.23
HR (bpm)	72.2 ± 19.4	75.0 ± 15.1	0.63
Pacemaker	4 (36.4)	5 (17.9)	0.24
DIA	0 (0.0)	1 (3.6)	1.0
CRT-D	0 (0.0)	0 (0.0)	-
Atrial fibrillation	7 (63.6)	17 (60.7)	1.0
Smoker	3 (27.3)	8 (29.6), N=27	1.0
Dyslipidemia	9 (81.8)	22 (78.6)	1.0
Diabetes (Type I)	0 (0.0)	0 (0.0)	-
Diabetes (Type II)	2 (18.2)	8 (28.6)	0.69
Hypertension	6 (54.6)	16 (57.1)	1.0
COBP	1 (9.1)	3 (10.7)	1.0
Sleep apnea syndrome	2 (18.2)	3 (10.7)	0.61
Arteriopathy	2 (18.2)	3 (10.7)	0.61
Stroke	1 (9.1)	3 (10.7)	1.0
NYHA			0.0016
I	0 (0.0)	2 (7.1)	
II	4 (36.4)	22 (78.6)	
III	5 (45.4)	4 (14.3)	
IV	2 (18.2)	0 (0.0)	
Coronaropathy > 50%	4 (36.4)	10 (35.7)	1.0
Poor mobility	4 (36.4)	3 (10.7)	0.082
Active endocarditis	0 (0.0)	0 (0.0)	-
Pulmonary hypertension	6 (54.6)	13 (46.4)	0.73
Previous cardiac surgery	5 (45.4)	4 (14.3)	0.085
Aortic valve replacement	*4*	*3*	
Aortic + mitral valve replacement	*0*	*1*	
Coronary artery bypass	*1*	*0*	
Hospitalisation for acute heart failure	4 (36.4)	8 (28.6)	0.71
Length of stay (days)	*6.5 (6 ; 9)*	*5.5 (4; 7)*	0.26
PNP	2 (18.2)	1 (3.6)	0.19
Carpal tunnel	2 (18.2)	8 (28.6)	0.69
Biceps tendon rupture	0 (0.0)	0 (0.0)	-
Inflammatory disease	0 (0.0)	0 (0.0)	-
Narrow lumbar canal	1 (9.1)	5 (17.9)	0.65
Narrow cervical canal	0 (0.0)	0 (0.0)	-
Deafness	0 (0.0)	0 (0.0)	-
Digestive trouble	0 (0.0)	1 (3.6)	1.0
Lower limb oedema	1 (9.1)	3 (10.7)	1.0
Orthostatic hypotension	1 (9.1)	6 (21.4)	0.65

Results are expressed as number (percentage), mean ± standard deviation or median (Q1; Q3); and respectively compared using Fisher's exact test, ANOVA or the Kruskal-Wallis non-parametric test.

Table S2. Echocardiographic data at baseline.

	No Tafamidis (N=11)		Tafamidis (N=28)		Comparison
	N non missing	Results	N non missing	Results	p-value
HR during echo (bpm)	11	72.5 ± 16.1	28	71.4 ± 13.8	0.84
BSA (m²)	11	1.8 ± 0.14	28	1.9 ± 0.12	0.14
Height (cm)	11	167 ± 4.7	28	169 ± 6.7	0.43
Telediastolic volume (ml)	11	73 (60; 100)	28	92 (68; 107)	0.26
Telesystolic volume (ml)	11	34 (27; 55)	28	37 (30; 45)	0.91
VES by volumes (ml)	11	69 (31; 51)	28	49 (40; 60)	0.10
VES by continuity equation (ml)	9	67 (59; 80)	26	65 (57; 74)	0.64
SVi (ml/m²)	9	34 (28; 43)	26	35 (30; 41)	0.79
LVEF (%)	11	54 (48; 60)	28	56 (51;62)	0.16
GLS average (%)	9	-10.2 (-12.3; -10.1)	27	-13.9 (-15.9; -11.2)	0.019
Apical sparing patters	9	9 (100.0)	27	24 (88.9)	0.56
Ratio apex/base >3	9	7 (77.8)	27	15 (55.6)	0.43
Myocardial index work (mmHg/%)	7	1037 (780; 1160)	25	1219 (963; 1347)	0.16
Global work efficiency (%)	7	90 (85; 92)	25	92 (87; 95)	0.14
LA Volume (ml/m²)	8	38 (32; 53)	22	48 (41;61)	0.15
LA area (cm²)	11	25 (22; 29)	28	26 (23; 29)	0.42
LA GLS S-CT (%)	6	-2.5 (-7; -2)	14	-4 (-5; -2)	0.83
LA GLS S-R (%)	6	13 (10; 21)	14	10 (8; 13)	0.51
Interauricular septum thickness (mm)	11	11 (9; 14)	27	10 (9; 12)	0.73
Atrial thrombus	11	1 (9.1)	28	1 (3.6)	0.49
Atrial thrombus at least one of the TTE	11	1 (9.1)	28	1 (3.6)	0.49
Interventricular septal thickness IVSd (mm)	11	16.0 (15.7; 18.0)	28	15.5 (13.5; 17.3)	0.20
LVEDD (mm)	11	45 (33; 50)	28	45 (39; 49)	0.79
Posterior wall thickness PWd (mm)	11	16.0 (14.0; 18.0)	28	13.9 (12.0; 16.7)	0.17
LV mass (g)	11	299 (237; 349)	28	261 (219; 313)	0.19
LV mass index (g/m²)	11	166 (137; 183)	28	138 (120; 169)	0.055
Relative wall thickness ratio (RWT)	10	0.72 (0.64; 1.0)	22	0.65 (0.50; 0.74)	0.17
LV remodeling	11		28		0.30
Normal		0 (0.0)		0 (0.0)	
Concentric remodeling		0 (0.0)		5 (17.9)	
Eccentric hypertrophy		0 (0.0)		0 (0.0)	
Concentric hypertrophy		11 (100.0)		23 (82.1)	
Deceleration time, DT (ms)	10	178 (145; 212)	28	204 (165; 278)	0.25
E Velocity (cm/s)	10	84 (72; 94)	28	89 (71; 120)	0.26
A Velocity (cm/s)	6	69 (44; 71)	18	70 (55; 85)	0.79
E/A	6	1.2 (0.74; 1.9)	18	1.5 (0.80; 2.2)	0.35
e' septal (cm/s)	9	4 (4; 5)	27	6 (5; 6)	0.041
e’ lateral (cm/s)	9	5 (4; 7)	27	7 (6; 9)	0.012
E/e’ mean	9	18.8 (11.0; 22.0)	27	14.0 (11.5; 16.5)	0.19
Vmax TR (m/s)	10	3.2 (2.9; 3.5)	21	2.9 (2.8; 3.0)	0.12
DP max IT (mmHg)	10	42 (32; 50)	21	34 (31; 37)	0.099

SPAP (mmHg)	10	53 (37; 60)	21	42 (36; 50)	0.19
RV diameter PSLAX (mm)	11	34 (29; 36)	27	34 (32; 37)	0.35
Pulmonary acceleration time (ms)	8	88 (69; 99)	21	101 (87; 133)	0.075
RVEDD (mm)	11	38 (32; 46)	26	39 (36; 43)	0.91
TAPSE (mm)	10	15 (15; 18)	28	20 (16; 24)	0.024
S' tricuspid (cm/s)	7	8 (7; 14)	24	11 (9; 13)	0.19
RA surface (cm^2)	10	18.2 (15.8; 21.8)	28	20.5 (16.2; 24.0)	0.47
GLS RV GS (%)	8	-9.7 (-15.6; -8.7)	17	-13.8 (-15.5; -11.1)	0.27
GLS RV SPL (%)	8	-12.9 (-19.6; -11.9)	17	-16.4 (-19.9; -12.8)	0.56
TR Grade	11		27		0.48
Trace		5 (45.4)		16 (59.3)	
Mild		5 (45.4)		9 (33.3)	
Moderate		1 (9.1)		1 (3.7)	
Severe		0 (0.0)		1 (3.7)	
MR Grade	11		28		0.70
Trace		4 (36.4)		12 (42.9)	
Mild		6 (54.5)		14 (50.0)	
Moderate		1 (9.1)		2 (7.1)	
Severe		0 (0.0)		0 (0.0)	
MS Grade	11		28		0.53
No		0 (0.0)		0 (0.0)	
Mild		11 (100.0)		27 (96.4)	
Moderate		0 (0.0)		1 (3.6)	
Severe		0 (0.0)		0 (0.0)	
Type of aortic valve	8		25		1.0
Tricuspid		7 (87.5)		23 (92.0)	
Biscuspid		1 (12.5)		2 (8.0)	
AR Grade	11		27		0.016
Trace		3 (27.3)		20 (74.1)	
Mild		7 (63.6)		5 (18.5)	
Moderate		1 (9.1)		2 (7.4)	
Severe		0 (0.0)		0 (0.0)	
AS Grade	11		27		0.070
Trace		7 (63.6)		23 (85.2)	
Mild		0 (0.0)		4 (14.8)	
Moderate		3 (27.3)		0 (0.0)	
Severe		1 (9.1)		0 (0.0)	
Peak velocity (m/s)	10	2.0 (1.6; 3.1)	27	1.4 (1.2; 1.6)	0.021
Aortic Max gradient (mmHg)	10	18.5 (10.0; 39.0)	27	7.0 (5.0; 9.6)	0.013
Aortic Mean gradient (mmHg)	10	9 (6; 20)	27	4 (3; 5)	0.0076
TVI AV (cm)	10	41.0 (31.9; 57.5)	26	28.0 (23.0; 32.7)	0.0094
TVI LVOT (cm)	9	18.4 (17.2; 19.3)	26	19.7 (16.0; 22.3)	0.46
Velocity ratio	9	0.40 (0.29; 0.57)	26	0.70 (0.55; 0.76)	0.030
LVOT diameter (cm)	11	21.7 (20.0; 24.0)	28	21.0 (20.0; 22.5)	0.64
Aortic valve area (AVA) (cm^2)	9	1.8 (1.1; 2.6)	26	2.4 (2.1; 2.7)	0.15
Indexed valvular area (cm^2/m^2)	9	1.0 (0.60; 1.3)	26	1.3 (1.1; 1.5)	0.096
Sinus Valsalva diameter (mm)	8	31.5 (29.5; 36.0)	25	35.0 (31.0; 38.0)	0.31
STJ diameter (mm)	4	28.5 (25.5; 35.5)	21	28.0 (25.0; 31.0)	0.66
Aortic diameter (mm)	6	30.5 (26.0; 33.0)	22	33.0 (28.0; 36.0)	0.43
Pericardial effusion	11	1 (9.1)	28	1 (3.6)	0.49

Results are expressed as number (percentage), mean ± standard deviation or median (Q1; Q3); and respectively compared using Fisher's exact test, ANOVA or the Kruskal-Wallis non-parametric test.

Table S3. Evolution of left ventricular mass at 1 year.

	No Tafamidis		Tafamidis		Comparison
	N	Median (Q1; Q3)	N	Median (Q1; Q3)	p-value [b]
SVi (ml/m²)					
D0	4	38.0 (29.5; 44.0)	11	34.0 (25.0; 41.0)	
D360	4	32.5 (28.5; 41.5)	11	33.0 (22.0; 42.0)	
Change from baseline	4	3.0 (-10.0; 6.5)	11	-2.0 (-5.0; 6.0)	0.74
p-value evolution [a]		1.0		0.78	
LV mass index (g/m²)					
D0	6	157.0 (137.0; 176.0)	15	135.0 (118.0; 167.0)	
D360	6	160.0 (148.0; 170.0)	15	148.0 (128.0; 173.0)	
Change from baseline	6	-5.5 (-25.0; 10.0)	15	4.0 (-4.0; 14.0)	0.24
p-value evolution [a]		0.62		0.25	

[a] *signed rank test at value for paired observations*
[b] *signed Kruskal-Wallis test at p value*

Table S4. Evolution of left ventricular mass: global evolution (GLMM)

	Effect	Coeff. ± SE	p-value
SVi (ml/m²)	Intercept	35.5 ± 2.7	-
	Time	0.0003 ± 0.010	0.98
	Tafamidis	0.80 ± 3.1	0.80
	Time x Tafamidis	0.0013 ± 0.012	0.91
	Intercept	172 ± 13.5	-
LV mass index (g/m²)	Time	-0.040 ± 0.050	0.84
	Tafamidis	-26.0 ± 15.8	0.10
	Time x Tafamidis	0.050 ± 0.061	0.41

Table S5. Evolution at 1 year of right ventricular function parameters

	No Tafamidis		Tafamidis		Comparison
	N	Median (Q1; Q3)	N	Median (Q1; Q3)	p-value [b]
RV diameter PSLAX (mm)					
D0	5	35.0 (32.0; 36.0)	14	33.5 (32.0; 36.0)	
D360	5	34.0 (33.0; 35.0)	14	34.0 (27.0; 38.0)	
Change from baseline	5	-1.0 (-1.0; 0)	14	1.0 (-3.0; 3.0)	0.54
p-value evolution [a]		0.50		0.91	
Pulmonary acceleration time (ms)					
D0	2	87.0 and 89.0	8	96.0 (89.0; 127.5)	
D360	2	65.0 and 101.0	8	105.5 (98.5; 116.5)	
Change from baseline	2	-22.0 and 12.0	8	9.5 (0.50; 20.5)	-
p-value evolution [a]		-		0.30	
RVEDD (mm)					
D0	5	36.0 (36.0; 39.0)	13	40.0 (37.0; 44.0)	
D360	5	40.0 (35.0; 44.0)	13	40.0 (39.0; 42.0)	
Change from baseline	5	1.0 (0.0; 1.0)	13	-1.0 (-3.0; 0.0)	0.15
p-value evolution [a]		0.50		0.42	
TAPSE (mm)					
D0	4	15.0 (12.5; 16.5)	13	19.0 (17.0; 24.0)	
D360	4	17.5 (12.0; 23.0)	13	20.0 (14.0; 21.0)	

Change from baseline	4	4.5 (-0.50; 6.5)	13	-2.0 (-3.0; 0.0)	0.099
p-value evolution [a]		0.38		0.077	
Tricuspid S' (cm/s)					
D0	3	8.0 (6.0; 11.0)	7	11.0 (10.0; 12.0)	
D360	3	11.0 (5.0; 12.0)	7	9.0 (8.0; 11.0)	
Change from baseline	3	1.0 (-1.0; 3.0)	7	-1.0 (-3.0; -1.0)	-
p-value evolution [a]		-		0.14	
RA surface (cm²)					
D0	5	18.5 (18.0; 20.0)	15	20.0 (16.0; 25.0)	
D360	5	18.0 (15.0; 18.0)	15	21.6 (15.2; 23.5)	
Change from baseline	5	0.0 (-5.0; 2.0)	15	0.20 (-3.0; 4.0)	0.46
p-value evolution [a]		0.62		0.70	
GLS RV GS (%)					
D0	1	-8.6	4	-12.6 (-16.5; -8.0)	
D360	1	-8.3	4	-15.7 (-5.4; -18.6)	
Change from baseline	1	0.30	4	0.85 (0.10; 3.3)	-
p-value evolution [a]		-		0.25	
GLS RV SPL (%)					
D0	1	-12.6	4	-20.9 (-6.3; -24.5)	
D360	1	-12.3	4	-10.8 (-19.1; -5.2)	
Change from baseline	1	0.30	4	1.7 (-0.15; 3.0)	-
p-value evolution [a]		-		0.38	

[a] *signed rank test at value for paired observations*
[b] *signed Kruskal-Wallis test at p value*

Table S6. Evolution of right ventricular function: global evolution (GLMM)

	Effect	Coeff. ± SE	p-value
RV diameter PSLAX (mm)	Intercept	32.9 ± 1.4	-
	Time	0.0052 ± 0.0049	0.29
	Tafamidis	1.7 ± 1.6	0.30
	Time x Tafamidis	-0.0080 ± 0.0058	0.17
Pulmonary acceleration time (ms)	Intercept	83.4 ± 8.6	-
	Time	-0.012 ± 0.035	0.73
	Tafamidis	22.7 ± 10.0	0.026
	Time x Tafamidis	-0.020 ± 0.041	0.63
RVEDD (mm)	Intercept	39.1 ± 1.7	-
	Time	-0.0013 ± 0.0061	0.83
	Tafamidis	-1.0 ± 2.0	0.60
	Time x Tafamidis	0.0078 ± 0.0073	0.29
TAPSE (mm)	Intercept	16.2 ± 1.5	-
	Time	0.0030 ± 0.0055	0.58
	Tafamidis	3.5 ± 1.8	0.050
	Time x Tafamidis	-0.0082 ± 0.0064	0.20
Tricuspid S' (cm/s)	Intercept	9.9 ± 0.99	-
	Time	0.0003 ± 0.0032	0.93
	Tafamidis	1.3 ± 1.1	0.25
	Time x Tafamidis	-0.0006 ± 0.0039	0.87
RA surface (cm²)	Intercept	19.7 ± 1.8	-
	Time	0.0028 ± 0.0063	0.65

	Tafamidis	-0.58 ± 2.1	0.78
	Time x Tafamidis	0.0019 ± 0.0074	0.80
GLS RV GS (%)	Intercept	-12.7 ± 1.5	-
	Time	0.0041 ± 0.0076	0.59
	Tafamidis	-1.3 ± 1.8	0.47
	Time x Tafamidis	0.0011 ± 0.0089	0.90
GLS RV SPL (%)	Intercept	-15.0 ± 2.1	-
	Time	0.0013 ± 0.011	0.90
	Tafamidis	-1.7 ± 2.5	0.51
	Time x Tafamidis	0.0063 ± 0.012	0.61

Table S7. Evolution of left atrium function at 1 year

	No Tafamidis		Tafamidis		Comparison
	N	Median (Q1; Q3)	N	Median (Q1; Q3)	p-value [b]
LA Volume (ml/m²)					
D0	2	36 and 62	9	49 (37; 57)	
D360	2	36 and 58	9	48 (41; 62)	
Change from baseline	2	0 and -4	9	4 (-4; 6)	-
p-value evolution [a]		-		0.59	
LA area (cm²)					
D0	5	25 (25; 25)	15	26 (23; 29)	
D360	5	26 (23; 30)	15	26 (24; 28)	
Change from baseline	5	0 (-2; 1)	15	0 (-3; 2)	0.93
p-value evolution [a]		1.0		1.0	
LA GLS S-CT (%)					
D0	1	-2	5	-4 (-5; -2)	
D360	1	-3	5	-4 (-5; -4)	
Change from baseline	1	-1	5	-1 (-1; 0)	-
p-value evolution [a]		-		0.50	
LA GLS S-R (%)					
D0	1	11	5	11 (8; 13)	
D360	1	8	5	9 (9; 10)	
Change from baseline	1	-3	5	0 (-4; 2)	-
p-value evolution [a]		-		1.0	

[a] *signed rank test at value for paired observations*
[b] *signed Kruskal-Wallis test at p value*

Table S8. Evolution of diastolic function at 1 year

	No Tafamidis		Tafamidis		Comparison
	N	Median (Q1; Q3)	N	Median (Q1; Q3)	p-value [b]
Deceleration time, DT (ms)					
D0	5	191 (145; 286)	15	182 (149; 260)	
D360	5	214 (171; 276)	15	179 (143; 198)	
Change from baseline	5	61 (1; 85)	15	-6 (-64; 11)	0.11
p-value evolution [a]		0.44		0.17	
E Velocity (cm/s)					
D0	5	73 (72; 94)	15	106 (75; 129)	
D360	5	79 (70; 117)	15	100 (80; 120)	
Change from baseline	5	3 (-12; 24)	15	3 (-17; 10)	0.57

p-value evolution [a]		1.0		0.55	
A Velocity (cm/s)					
D0	3	68 (38; 99)	6	73 (69; 87)	
D360	3	40 (32; 99)	6	74 (64; 87)	
Change from baseline	3	-6 (-28; 0)	6	3 (-19; 17)	-
p-value evolution [a]		-		1.0	
E/A					
D0	3	0.74 (0.62; 1.9)	6	1.4 (0.80; 1.9)	
D360	3	1.6 (0.44; 2.3)	6	1.2 (1.1; 1.5)	
Change from baseline	3	0.45 (-0.30; 0.98)	6	-0.005 (-0.27; 0.61)	-
p-value evolution [a]		-		1.0	
e' septal (cm/s)					
D0	3	4.0 (3.0; 6.0)	12	6.0 (5.5; 7.5)	
D360	3	3.0 (3.0; 4.0)	12	5.0 (5.0; 6.5)	
Change from baseline	3	-1.0 (-3.0; 1.0)	12	-1.0 (-1.0; 0.0)	-
p-value evolution [a]		-		0.055	
e' lateral (cm/s)					
D0	4	4.5 (3.5; 6.5)	13	7.0 (7.0; 9.0)	
D360	4	3.5 (3.0; 5.0)	13	7.0 (6.5; 9.0)	
Change from baseline	4	0.0 (-1.0; 1.0)	13	0.0 (-1.0; 1.0)	0.23
p-value evolution [a]		0.25		0.73	
E/e' mean					
D0	3	11 (9; 22)	12	15 (12; 17)	
D360	3	20 (13; 23)	12	16 (13; 19)	
Change from baseline	3	11 (-9; 12)	12	3 (0; 3)	-
p-value evolution [a]		-		0.11	
DP max IT (mmHg)					
D0	5	45 (32; 50)	11	37 (31; 44)	
D360	5	41 (36; 41)	11	33 (23; 43)	
Change from baseline	5	-1 (-11; 9)	11	-3 (-20; 1)	0.50
p-value evolution [a]		0.63		0.11	

[a] *signed rank test at value for paired observations*
[b] *signed Kruskal-Wallis test at p value*

Table S9. Evolution of diastolic function: global evolution (GLMM)

	Effect	Coeff. ± SE	p-value
Deceleration time, DT (ms)	Intercept	201 ± 20.3	-
	Time	0.0095 ± 0.072	0.19
	Tafamidis	16.7 ± 23.6	0.48
	Time x Tafamidis	-0.15 ± 0.085	0.087
E Velocity (cm/s)	Intercept	83.2 ± 7.6	-
	Time	-0.0043 ± 0.027	0.87
	Tafamidis	14.2 ± 8.8	0.11
	Time x Tafamidis	-0.0049 ± 0.032	0.88
A Velocity (cm/s)	Intercept	72.0 ± 9.0	-
	Time	-0.066 ± 0.036	0.073
	Tafamidis	-3.3 ± 10.4	0.75
	Time x Tafamidis	0.088 ± 0.043	0.045
E/A	Intercept	1.3 ± 0.27	-

	Time	-0.00004 ± 0.0011	0.97
	Tafamidis	0.30 ± 0.31	0.34
	Time x Tafamidis	-0.0007 ± 0.0013	0.59
e' septal (cm/s)	Intercept	4.5 ± 0.55	-
	Time	-0.0029 ± 0.0021	0.18
	Tafamidis	1.5 ± 0.62	0.015
	Time x Tafamidis	0.0011 ± 0.0024	0.65
e' lateral (cm/s)	Intercept	5.7 ± 0.74	-
	Time	-0.0002 ± 0.0026	0.93
	Tafamidis	2.6 ± 0.85	0.0034
	Time x Tafamidis	-0.0014 ± 0.0030	0.64
E/e' mean	Intercept	17.4 ± 1.7	-
	Time	0.0079 ± 0.00065	0.23
	Tafamidis	-3.2 ± 1.9	0.095
	Time x Tafamidis	-0.0042 ± 0.0073	0.57
DP max IT (mmHg)	Intercept	40.8 ± 3.0	-
	Time	-0.0087 ± 0.010	0.40
	Tafamidis	-7.7 ± 3.7	0.037
	Time x Tafamidis	0.00052 ± 0.013	0.97

Table S10. Search for deterioration at 1 year of valvulopathies in the group with Tafamidis (N=20 patients with values at days 0 and 360)

	Baseline (D0) n (%)	D360 n (%)	Evolution p-value [a]
TR Grade			0.48
Trace	9 (45.0)	7 (35.0)	
Mild	9 (45.0)	10 (50.0)	
Moderate	2 (10.0)	3 (15.0)	
Severe	0 (0.0)	0 (0.0)	
MR Grade			0.18
Trace	9 (45.0)	6 (30.0)	
Mild	9 (45.0)	10 (50.0)	
Moderate	2 (10.0)	4 (20.0)	
Severe	0 (0.0)	0 (0.0)	
MS Grade			-
No	0 (0.0)	0 (0.0)	
Mild	20 (100.0)	20 (100.0)	
Moderate	0 (0.0)	0 (0.0)	
Severe	0 (0.0)	0 (0.0)	
AR Grade (N=19)			0.65
Trace	13 (68.4)	12 (63.2)	
Mild	5 (26.3)	7 (36.8)	
Moderate	1 (5.3)	0 (0.0)	
Severe	0 (0.0)	0 (0.0)	
AS Grade (N=19)			1.0
Trace	16 (80.0)	16 (80.0)	
Mild	2 (10.0)	1 (5.0)	
Moderate	1 (5.0)	3 (15.0)	
Severe	1 (5.0)	0 (0.0)	
